# ABO blood group A transferase and its codon 69 substitution enzymes synthesize FORS1 antigen of FORS blood group system

**DOI:** 10.1038/s41598-019-46029-7

**Published:** 2019-07-04

**Authors:** Miyako Yamamoto, Maria Cristina Tarasco, Emili Cid, Hidetomo Kobayashi, Fumiichiro Yamamoto

**Affiliations:** 1 Laboratory of Immunohematology and Glycobiology, Josep Carreras Leukaemia Research Institute (IJC), Campus Can Ruti, Camí de les Escoles, Badalona, Barcelona 08916 Spain; 2 Biologia Molecolare, Università degli Studi di Parma, Parma, 43121 Italy; 3 Program of Predictive and Personalized Medicine of Cancer (PMPPC), Institut d’Investigació Germans Trias i Pujol (IGTP), Campus Can Ruti, Camí de les Escoles, Badalona, Barcelona 08916 Spain; 4 Laboratory of Molecular Microbiological Science, Faculty of Pharmaceutical Sciences, Hiroshima International University, Kure, Hiroshima 737-0112 Japan

**Keywords:** Glycobiology, Immunochemistry, Glycobiology, Immunogenetics, Immunogenetics

## Abstract

Human histo-blood group A transferase (AT) catalyzes the biosynthesis of oligosaccharide A antigen important in blood transfusion and cell/tissue/organ transplantation. This enzyme may synthesize Forssman antigen (FORS1) of the FORS blood group system when exon 3 or 4 of the AT mRNA is deleted and/or the LeuGlyGly tripeptide at codons 266–268 of AT is replaced by GlyGlyAla. The Met69Ser/Thr substitutions also confer weak Forssman glycolipid synthase (FS) activity. In this study, we prepared the human AT derivative constructs containing any of the 20 amino acids at codon 69 with and without the GlyGlyAla substitution, transfected DNA to newly generated COS1(B3GALNT1 + A4GALT) cells expressing an enhanced level of globoside (Gb4), the FS acceptor substrate, and immunologically examined the FORS1 expression. Our results showed that all those substitution constructs at codon 69 exhibited FS activity. The combination with GlyGlyAla significantly increased the activity. The conserved methionine residue in the *ABO*, but not *GBGT1*, gene-encoded proteins may implicate its contribution to the separation of these genes in genetic evolution. Surprisingly, with increased Gb4 availability, the original human AT with the methionine residue at codon 69 was also demonstrated to synthesize FORS1, providing another molecular mechanism of FORS1 appearance in cancer of ordinary FORS1-negative individuals.

## Introduction

**Figure 1:**
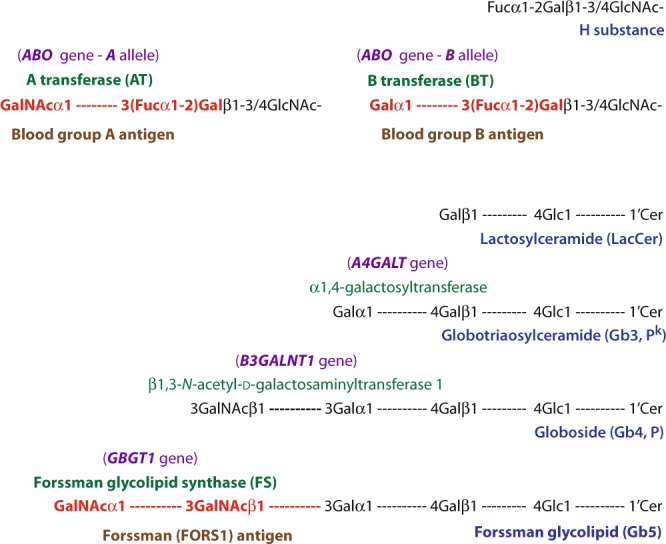
Schematic representation of biosynthetic pathways of blood group A, B, and FORS1 antigens. The biosynthetic pathways of blood group A, B, and FORS1 antigens and related glycans are schematically shown. The names of genes, transferases, glycans, and antigens are shown in purple, green, blue, and brown colors, respectively. Important genes/transferases/glycans are shown in bold type. Immunodominant epitopes of A, B, and FORS1 are shown in red color and bold type.

Human blood group ABO system (ABO) consists of A and B antigens expressed on red blood cells (RBCs) and the antibodies against these antigens in the sera of individuals who do not express the antigen(s) (Landsteiner’s Law). The A and B antigens are oligosaccharides with the following chemical structures: GalNAcα1-3(Fucα1-2)Gal- (A antigen) and Galα1-3(Fucα1-2)Gal- (B antigen)^1,2^. Functional A and B alleles at the *ABO* genetic locus encode A and B transferases (AT and BT), which catalyze the last biosynthetic reactions of A and B antigens by transferring an *N*-acetyl-d-galactosamine (GalNAc) and galactose from nucleotide-sugars UDP-GalNAc and UDP-galactose, respectively, to the common precursor substrate H substance (Fucα1-2Gal-). The Forssman system (FORS) is another blood group system with oligosaccharide Forssman antigen (FORS1) and its antibodies^3^. *Homo sapiens* being a Forssman antigen-negative species^4^, ordinary individuals are FORS1-negative (FORS1−) and do not express FORS1 antigen^3,5^. However, there are FORS1-positive (FORS1+) individuals expressing FORS1 although the frequency is extremely low in the human population (rs375748588 SNP, MAF/Minor Allele Count: T = 0.000008/1 (ExAC), T = 0.00008/1 (GO-ESP), T = 0.00005/6 (TOPMED)). The International Society of Blood Transfusion (ISBT) numbered the ABO and FORS systems to be 001 and 031, respectively. Forssman glycolipid synthase (FS) encoded by the functional allele at the *GBGT1* gene^5,6^ catalyzes the last biosynthetic step of FORS1-carrying penta-saccharide Forssman glycolipid (Gb5: GalNAcα1-3GalNAcβ1-3Galα1-4Galβ1-4Glcβ1-1′Cer) from the precursor globoside (Gb4: GalNAcβ1-3Galα1-4Galβ1-4Glcβ1-1′Cer) by transferring a GalNAc^7^. Biosynthetic pathways of blood group A, B, and FORS1 antigens are schematically shown in Fig. 1.

In 1990, we cloned the blood group A, B, and O allelic cDNAs, determined their nucleotide sequences, and correlated their sequences with the A/B antigen expression. We found that A allele-encoded AT and B allele-encoded BT are the same in size, but there are 4 amino acid substitutions between them^8,9^. They are arginine (Arg), glycine (Gly), leucine (Leu), and Gly in AT, and Gly, serine (Ser), methionine (Met), and alanine (Ala) in BT at codons 176, 235, 266, and 268. We prepared 14 AT-BT chimeras that were different at those 4 positions, having either amino acid of AT or BT, transfected DNA from those constructs to human carcinoma of uterus HeLa cells expressing H substance, the precursor substrate for AT/BT, and examined the appearance of cell-surface A and/or B antigens. We were able to demonstrate that the amino acids at codons 266 and 268 are crucial on sugar specificity and enzymatic activity, whereas those at codons 235 and 176 exerted slight and no effect, respectively^10^. We also identified a single nucleotide deletion (261delG) in the majority of nun-functional O alleles^9^ and a single inactivating glycine-to-arginine substitution at codon 268 (Gly268Arg) in the AT background in some O alleles^11^.

We also investigated the molecular genetic basis of Forssman antigen negativity in humans, and found 2 inactivating missense mutations; Ser and Arg at codons 230 and 296, respectively, in the human *GBGT1* gene-encoded non-functional protein^12^. Using the DNA transfection assays of African green monkey kidney COS1 cells expressing Gb4 as recipients, we showed that the substitution of the Ser to Gly or the Arg to glutamine (Gln), corresponding amino acids of the FSs in Forssman antigen-positive mice and some other species, rendered the human protein with weak FS activity whereas the reversion of both conferred strong FS activity. The Arg269Gln reversion was later found to be responsible for the activation of *GBGT1* gene in rare FORS1 + individuals^3^.

During species evolution, the GlyGlyAla tripeptide sequence at codons corresponding to 266-268 of human AT/BT has been well conserved in the majority of *GBGT1* gene-encoded FSs^13^. We realized that mouse *ABO* gene-encoded *cis*-AB transferase also possesses the GlyGlyAla sequence^14^. We then examined whether or not this murine enzyme might also exhibit the FS activity by transfecting the expression construct into COS1(B3GALNT1) cells expressing transduced human *B3GALNT1* gene cDNA encoding β1,3-*N*-acetyl-d-galactosaminyltransferase 1 to increase Gb4 content. We observed the appearance of FORS1, whereas human *cis*-AB transferase with LeuGlyAla tripeptide did not exhibit such activity^15^. We also detected weak FS activity of the modified human AT, whose LeuGlyGly was replaced with GlyGlyAla. Those results demonstrated that the GlyGlyAla sequence is important for the FS activity, but that this substitution is not sufficient to provide human AT with full FS activity. We, therefore, initiated the search for additional molecular mechanisms that would allow ATs to catalyze the FORS1 biosynthesis. We found that the deletion of exon 3 or 4, but not of exon 2 or 5, of human AT transcripts bestowed moderate FS activity^16^, indicating that the A allele is inherently capable of producing a protein with FS activity. Because altered RNA splicing is frequent in cancer, this mechanism may explain, at least partially, the FORS1 appearance in some cancer cells/tissues of regular individuals in the Forssman antigen-negative human species^17–23^. Additionally, we also serendipitously observed that the methionine to serine/threonine substitutions at codon 69 (Met69Ser/Thr) conferred weak FS activity^24^. Furthermore, co-introduction of one of those deletions or Met69Ser/Thr substitutions with the LeuGlyGly266-268GlyGlyAla substitution strengthened FS activity, possibly by the synergistic effects of altered intra-Golgi localization and/or protein conformation by the former and modified enzymatic specificity by the latter.

In the present study we have generated the COS1(B3GALNT1 + A4GALT) cells expressing transduced human *A4GALT* gene cDNA encoding α1,4-galactosyltransferase, in addition to the human *B3GALNT1* gene cDNA encoding β1,3-*N*-acetyl-d-galactosaminyltransferase 1, in order to further improve the detection sensitivity of FS activity. We have also substituted the methionine residue at codon 69 of the human AT by any one of the other 19 amino acids, with and without the GlyGlyAla substitution, and examined the FS activity of those encoded proteins by DNA transfection and immunological detection of FORS1. Here, we report differential FS activity of the ATs with a missense mutation at codon 69. We also present our surprising finding that the original human AT may also catalyze the FORS1 biosynthesis under certain circumstances.

## Results

### Methionine is conserved at codons corresponding to 69 of human AT/BT of the *ABO* gene-encoded proteins in a variety of species, but not of the *GBGT1* gene-encoded proteins

The *ABO*, *GBGT1*, *A3GALT2*, *GGTA1*, and *GLT6D1* genes are paralogous genes evolved from the same ancestral gene^13,25,26^. While it remains to be determined whether the *GLT6D1* gene-encoded proteins may have glycosyltransferase activity or not, the functional genes in the first 4 categories encode proteins with glycosylation functions: ATs/BTs, FSs, isoglobotriaosylceramide synthases (iGb3Ss) to synthesize isoglobotriaosylceramide (iGb3: Galα1-3Galβ1-4Glcβ1-1’Cer), and α1,3-galactosyltransferases (GTs) to synthesize the α1,3-galactosyl epitope (α-Gal-epitope: Galα1-3Galβ1-4GlcNAcβ-). All those glycosyltransferase genes have sequence homology in the last 2 coding exons, whereas the genes encoding ATs/BTs and FSs are homologous in the last 3 coding exons.

Codon 69 of human AT/BT is located at the beginning of exon 5, the 3^rd^ coding exon from the last. In order to compare amino acid residues at codons corresponding to 69 of human AT/BT, we used the CLUSTALW aligned amino acid sequences of proteins encoded by the *ABO* and *GBGT1* gene orthologs from various species (Gene Tree: ENSGT00950000182858). As shown in Table 1, they are methionines in the *ABO* genes in a variety of species. Although there are species with other amino acids at that position, they are a minority. Because the methionine residue is present over phylogenetic boundaries from amphibians to mammals and to primates, it seems to be the prototype in the *ABO* genes. On the other hand, the methionine residue was rarely present at that position in the *GBGT1* genes although it was coincidentally found in mangrove killifish. Therefore, the methionine residue at that position differentiates well between the *ABO* and *GBGT1* gene-encoded proteins.

**Table 1:** Species possessing the methionine residue at codons corresponding to 69 of the human AT/BT in the *ABO* and *GBGT1* gene-encoded proteins.

**(A). Species possessing the methionine in the** ***ABO*** **gene-encoded proteins**
*Xenopus* frog, painted turtle, Agassiz’s desert tortoise, elephant, horse, donkey, cat, red fox, microbat, dog, dingo, polar bear, American black bear, panda, rat, Chinese hamster, Northern American deer mouse, Upper Galilee mountains blind mole rat, kangaroo rat, rabbit, pika, mouse lemur, Coquerel’s sifaka, tarsier, capuchin, marmoset, crab-eating macaque, macaque, sooty mangabey, drill, vervet African green monkey (vervet-AGM), olive baboon, Black snub-nosed monkey, bonobo, and human
**(B). Species possessing the methionine in the** ***GBGT1*** **gene-encoded proteins**
Mangrove killifish

### COS1(B3GALNT1 + A4GALT) cells expressing human *B3GALNT1* gene-encoded β1,3-*N*-acetyl-d-galactosaminyltransferase 1 and human *A4GALT* gene-encoded α1,4-galactosyltransferase were generated

**Figure 2:**
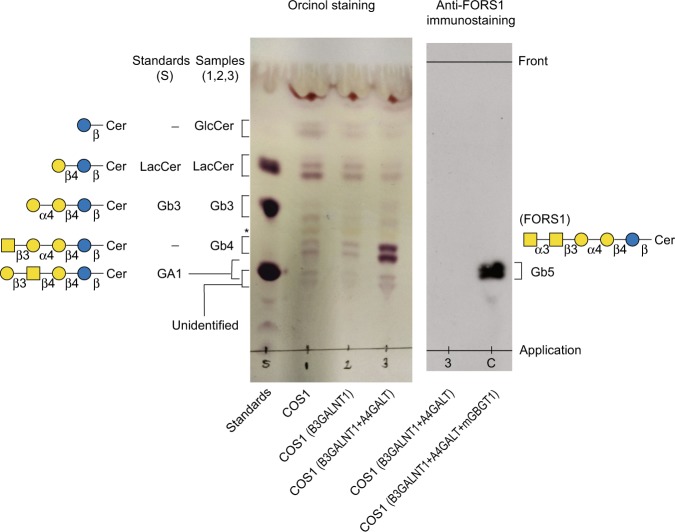
Thin-layer chromatography (TLC) analysis of neutral glycosphingolipids isolated from COS1 and derivative cells. The orcinol staining panel shows nGSLs as purple bands. Lane S corresponds to the standards: lactosylceramide (LacCer), globotriaosylceramide (Gb3) and gangliotetraosylceramide (asialo-GM1, GA1). Samples are nGSLs isolated from COS1, COS1(B3GALNT1), and COS1(B3GALNT1 + A4GALT) cells for lanes 1-3, respectively. The bands are marked, and their simplified molecular structures are shown on the left. GlcCer is glucosylceramide, Gb4 corresponds to globoside, and the asterisk indicates an orange band of a lipid not containing glycans. Another TLC plate containing nGSLs from COS1(B3GALNT1 + A4GALT) cells (lane 3) and nGSLs from those cells transduced with mouse *GBGT1* gene cDNA encoding FS, COS1(B3GALNT1 + A4GALT + mGBGT1) (lane C), was subjected to direct immunostaining using anti-FORS1 antibody. The FORS1-carrying Gb5 bands are marked, together with the simplified molecular structure of Gb5 on the right. The application locations and final solvent front are also indicated.

Previously, we prepared COS1(B3GALNT1) cells by transduction of African green monkey kidney COS1 cells with the retroviral vector to express human *B3GALNT1* gene cDNA encoding β1,3-*N*-acetyl-d-galactosaminyltransferase 1^15^. Use of those cells as a host for DNA transfection somewhat improved the detection sensitivity of FS activity^15^. However, thin-layer chromatography (TLC) analysis of the neutral glycosphingolipid (nGSL) fraction isolated from COS1(B3GALNT1) cells revealed low globoside (Gb4) content in spite of that manipulation, as shown in lane 2 of the left panel in Fig. 2. It also showed that lactosylceramide (LacCer) was abundant but globotriaosylceramide (Gb3) was not. Because LacCer is the precursor of Gb3, we hypothesized that weak activity of α1,4-galactosyltransferase, which is also called as Gb3 synthase, might be the rate-limiting factor of Gb4 synthesis. Due to insufficient Gb3 substrates, β1,3-*N*-acetyl-d-galactosaminyltransferase 1 may not synthesize Gb4 efficiently even when the transduced gene is expressed in those cells. Therefore, we further transduced COS1(B3GALNT1) cells with the retroviral vector encoding human α1,4-galactosyltransferase, and generated COS1(B3GALNT1 + A4GALT) cells expressing both β1,3-*N*-acetyl-d-galactosaminyltransferase 1 and α1,4-galactosyltransferase. As shown in lane 3 of the left panel in Fig. 2, the newly created cells produced an enhanced level of Gb4. However, we also observed weaker bands of larger sizes than Gb4. In order to exclude the possibility that those extra bands are of Forssman glycolipid (Gb5), we performed direct immunostaining of nGSLs from COS1(B3GALNT1 + A4GALT) cells on TLC using anti-FORS1 antibody. For comparison, nGSLs isolated from COS1(B3GALNT1 + A4GALT) cells that were transduced with mouse *GBGT1* gene cDNA encoding functional FS, COS1(B3GALNT1 + A4GALT + mGBGT1) cells, was applied in parallel. Results of immunostaining are shown in the right panel of Fig. 2. Gb5 carrying FORS1 antigen was found present in nGSLs isolated from COS1(B3GALNT1 + A4GALT + mGBGT1) cells (lane C), but not from COS1(B3GALNT1 + A4GALT) cells (lane 3). We, therefore, concluded that COS1(B3GALNT1 + A4GALT) cells expressing increased Gb4 might be useful to detect FS activity with improved sensitivity.

### Human A transferase (AT) expression constructs containing any amino acid substitution at codon 69 with and without the LeuGlyGly266-268GlyGlyAla substitution were prepared

We performed primer-mediated *in vitro* mutagenesis to prepare human AT amino acid substitution constructs at codon 69 with and without the LeuGlyGly266-268GlyGlyAla substitution. We obtained many, but not all, of the constructs by two-round polymerase chain reactions (PCRs) using degenerate oligonucleotide primers. To obtain those missing, we utilized specific oligonucleotide primers designed to introduce the corresponding mutations. In the end, we successfully obtained all the forty constructs with any one of 20 possible amino acids at codon 69 and 2 constructs with the translation termination codon (Ter) at that position. Amino acid residues at important codons and other characteristics of the expression constructs used in this study are listed in Table 2.

**Table 2:** Amino acid residues at important codons and other changes of the expression constructs used in the study.

Construct Name	Amino acid residue at codon				Additional change(s)
	69	266	267	268	
H_ABO-A	Met	Leu	Gly	Gly	
H_ABO-B	Met	Met	Gly	Ala	Arg176Gly, Gly235Ser
M_GBGT1	Leu	Gly	Gly	Ala	Different gene Many more
M_ABO-AB	Asn	Gly	Gly	Ala	Different gene Many more
H_ABO-A(delEx3 & GlyGlyAla)	Met	Gly	Gly	Ala	Exon 3 deletion
H_ABO-A(delEx4 & GlyGlyAla)	Met	Gly	Gly	Ala	Exon 4 deletion
H_ABO-A(Met69Xxx)	Xxx (Various)	Leu	Gly	Gly	
H_ABO-A(Met69Xxx & GlyGlyAla)	Xxx (Various)	Gly	Gly	Ala	

### Various amino acid substitutions at codon 69 of human AT conferred FS activity of differential strength

Using COS1(B3GALNT1 + A4GALT) cells as recipients, transient transfection experiments were performed with DNA from the AT or its mutant constructs and DNA from the pEGFP plasmid vector expressing enhanced green fluorescent protein (GFP), followed by monitoring GFP-positive (GFP+) cells under the fluorescence microscope to estimate transfection efficiency and immunocytochemical detection of the FORS1 expression.

**Figure 3:**
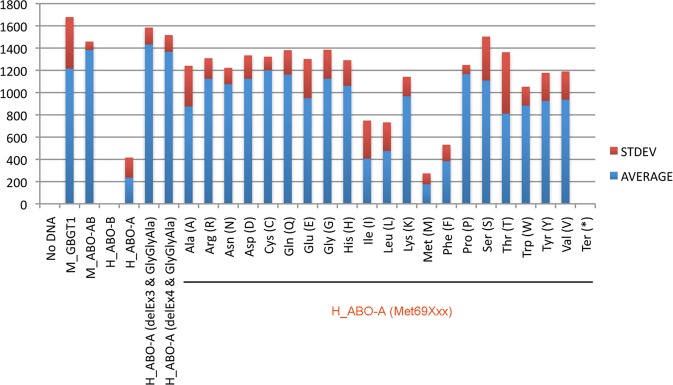
FORS1 antigen expression after DNA transfection of a variety of human AT amino acid substitution constructs at codon 69 into COS1(B3GALNT1 + A4GALT) cells analyzed by immunocytochemistry. The averages and standard deviations of FORS1 + cells are shown of the cells transfected with human AT amino acid substitution constructs at codon 69, H_ABO-A(Met69Xxx). “No DNA” without sample DNA was used as a negative control, and M_GBGT1 and M_ABO-AB constructs encoding mouse FS and *cis*-AB transferases, respectively, were used as positive controls. The H_ABO-A and H_ABO-B encode human AT and BT, respectively. The H_ABO-A(delEx3 & GlyGlyAla) and H_ABO-A(delEx4 & GlyGlyAla) represent the constructs with the deletion of exon 3 or 4 in combination with the LeuGlyGly266-268GlyGlyAla substitution, respectively. All the amino acid substitution constructs at codon 69 are shown by the substituted amino acid names in the one-letter and three-letter codes.

The results of AT substitution constructs containing an amino acid substitution at codon 69 without the LeuGlyGly266-268GlyGlyAla substitution are shown in Fig. 3. The positive control constructs of mouse *GBGT1* and *ABO* genes, M_GBGT1 and M_ABO-AB, respectively, exhibited strong FORS1 antigen expression, whereas the “No DNA” control presented a negative result. The B allele construct of the human *ABO* gene, H_ABO-B, also gave a negative result. As previously reported^16^, the human AT constructs containing the deletion of exon 3 or 4 in combination with the LeuGlyGly266-268GlyGlyAla substitution, H_ABO-A(delEx3 & GlyGlyAla) and H_ABO-A(delEx4 & GlyGlyAla), strongly expressed FORS1 antigen. The human AT constructs containing an amino acid substitution at codon 69 exhibited differential expression of FORS1 antigen, depending on which amino acid substituted. Surprisingly, the original A allele construct of the human *ABO* gene, H_ABO-A, as well as the methionine construct, H_ABO-A(Met69Met), having the same amino acid sequence as H_ABO-A construct also exhibited the FORS1 expression although the activity was the lowest. The H_ABO-A(Met69Ile) and H_ABO-A(Met69Leu) constructs with aliphatic amino acids, isoleucine and leucine, respectively, also showed low FS activities, whereas the construct with the translation termination codon, H_ABO(Met69Ter), expressed no FORS1 antigen.

**Figure 4:**
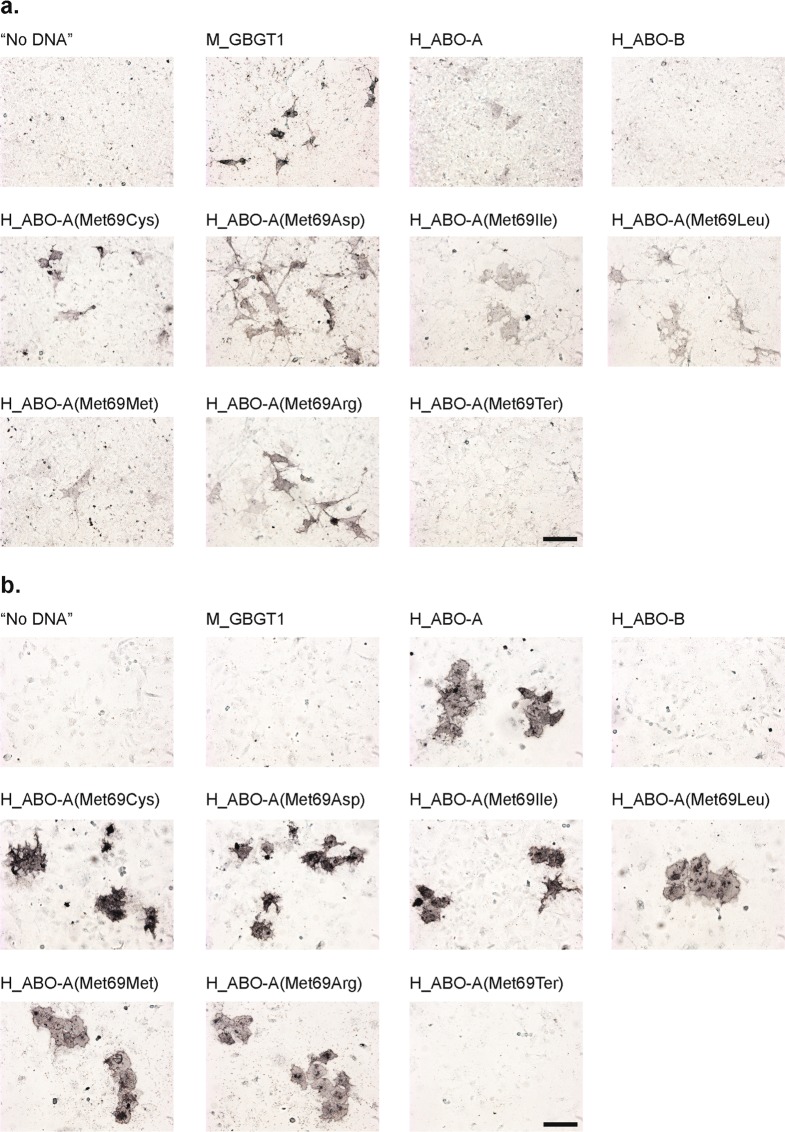
Microphotographs of COS1(B3GALNT1 + A4GALT) cells immunostained with the anti-FORS1 antibody and those of HeLa(FUT2) cells immunostained with anti-A antibodies after DNA transfection with the selected human AT derivative constructs. (**a**) COS1(B3GALNT1 + A4GALT) cells were transfected with the selected human AT derivative constructs and immunostained with clone FOM-1 rat anti-FORS1 antibody. The cells were next incubated with the biotinylated anti-rat IgG + IgM(H + L) secondary antibody followed by the Vectastain ABC reagents treatment and DAB color development. The “No DNA” corresponds to cells without DNA transfection. The constructs transfected are: M_GBGT1, H_ABO-A, H_ABO-B, H_ABO-A(Met69Cys), H_ABO-A(Met69Asp), H_ABO-A(Met69Ile), H_ABO-A(Met69Leu), H_ABO-A(Met69Met), H_ABO-A(Met69Arg), and H_ABO-A(Met9Ter). The transfected DNA is indicated above each image. All the images were taken with the same settings. The bar corresponds to 100 μm, (**b**) HeLa(FUT2) cells were transfected with the same set of human AT derivative constructs and immunostained with mouse anti-A BioClone antibodies, next with the biotinylated goat anti-mouse IgM secondary antibody, followed by the ABC reagents incubation and DAB color development. All the images were taken with the same settings. The bar corresponds to 100 μm.

In order to take microphotographs of cells immunostained with anti-FORS1 antibody after DNA transfection, we performed another immunocytochemistry experiment of selected constructs, using chambered cell culture slides. Figure 4a shows the results of human AT derivative constructs containing Cys, Asp, Ile, Leu, Met, Arg, and Ter at codon 69, H_ABO-A(Met69Cys), H_ABO-A(Met69Asp), H_ABO-A(Met69Ile), H_ABO-A(Met69Leu), H_ABO-A(Met69Met), H_ABO-A(Met69Arg), and H_ABO-A(Met69Ter), as well as mouse FS and human AT and BT constructs, M_GBGT1, H_ABO-A, and H_ABO-B, respectively.

### Amino acid substitutions at codon 69 of the human AT did not affect much of the AT activity

The AT activity was examined of the human AT amino acid substitution constructs at codon 69 by DNA transfection into HeLa(FUT2) cells followed by the immunocytochemical detection of A antigen. Strong AT activity was present for all those human AT constructs with an amino acid substitution at codon 69 (data not shown). In order to take the microphotographs of HeLa(FUT2) cells immunostained with anti-A antibodies after DNA transfection, we also performed another immunocytochemistry experiment using chambered cell culture slides. The same set of constructs used above with COS1(B3GALNT1 + A4GALT) cells were transfected to HeLa(FUT2) cells. Results are shown in Fig. 4b.

### Enhanced intra-cellular globoside (Gb4) availability of the recipient cells of DNA transfection significantly influences the observed FS activity

**Figure 5:**
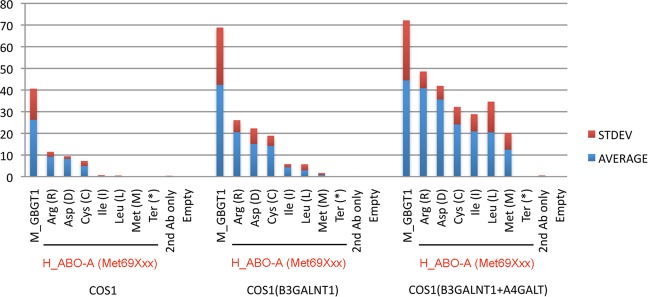
Expression of FORS1 antigen after DNA transfection of the selected human AT amino acid substitution constructs at codon 69 into COS1, COS1(B3GALNT1), and COS1(B3GALNT1 + A4GALT) cells analyzed by cytometry. African green monkey kidney COS1 cells and their derivative sublines of COS1(B3GALNT1) and COS1(B3GALNT1 + A4GALT) cells were separately transfected with the selected human AT derivative constructs, immunostained with the anti-FORS1 antibody followed by cytometry. The averages and standard deviations of FORS1 + cell percentages among the gated cells are shown of individual constructs in a bar graph. The secondary antibody only “2nd Ab only” and “Empty” samples were used as negative controls.

In addition to the immunocytochemical detection, we have also examined the expression of the FORS1 antigen by cytometry, and obtained more quantitative data. In this set of experiments, we utilized COS1 cells and two related sublines of cells, COS1(B3GALNT1) and COS1(B3GALNT1 + A4GALT). We used above-selected 7 human AT derivative constructs, together with mouse FS construct, M_GBGT1, as a positive control. DNA from pLL3.7-mRFP plasmid vector encoding monomeric red fluorescent protein (mRFP) was co-transfected to examine the transfection efficiency. After immunostaining of DNA-transfected cells with anti-FORS1 antibody, the total numbers of gated cells, mRFP-positive (mRFP+) cells, and the Alexa Fluor 647 dye-positive (AF647+) cells expressing FORS1 antigen were counted, and the FORS1+ cell percentages were then calculated after normalization. Results are shown in Fig. 5. Highest FORS1 antigen expression was generally observed of the COS1(B3GALNT1 + A4GALT) cells, next with COS1(B3GALNT1) cells, and the lowest with COS1 cells, as anticipated from the abundance of acceptor substrate Gb4. The variations in FS activity among the constructs were more evident when the Gb4 availability was restricted. On the contrary, FS activity of the original AT encoded by H_ABO-A(Met69Met) was obvious when sufficient Gb4 substrates were available in the recipient cells of DNA transfection.

### FS activity of the codon 69 amino acid substitution constructs was significantly enhanced by the concomitant LeuGlyGly266-268GlyGlyAla substitution

**Figure 6:**
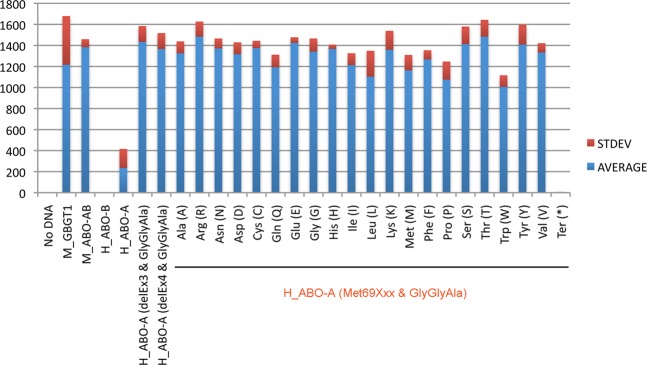
FORS1 antigen expression after DNA transfection of a variety of human AT amino acid substitution constructs at codon 69 with the LeuGlyGly266-268GlyGlyAla substitution into COS1(B3GALNT1 + A4GALT) cells analyzed by immunocytochemistry. The human AT amino acid substitution constructs at codon 69 with the LeuGlyGly266-268GlyGlyAla substitution, H_ABO-A(Met69Xxx & GlyGlyAla), were examined for the FS activity. See the Fig. 3 legend for the detailed information of those constructs except the LeuGlyGly266-268GlyGlyAla substitution was added.

The results of immunocytochemistry experiments after transfection of DNA from AT substitution constructs containing an amino acid substitution at codon 69 and the LeuGlyGly266-268GlyGlyAla substitution into COS1(B3GALNT1 + A4GALT) cells are shown in Fig. 6. Similar to the results of DNA transfection with the human AT constructs without the LeuGlyGly266-268GlyGlyAla substitution described above, the positive and negative controls presented anticipated positive and negative results. In addition, the amino acid substitution constructs at codon 69 exhibited substantially higher FORS1 antigen expression when combined with the LeuGlyGly266-268GlyGlyAla substitution, possibly due to the synergistic effects previously reported of the AT constructs containing the deletion of exon 3 or 4, or Met69Ser/Thr substitution in combination with the LeuGlyGly266-268GlyGlyAla substitution^16,24^. However, the Ter construct, H_ABO-A(Met69Ter & GlyGlyAla), did not present any FS activity even with the LeuGlyGly266-268GlyGlyAla substitution.

## Discussion

In our previous studies utilizing COS1(B3GALNT1) cells as DNA transfection recipients, we showed that the Met69Ser/Thr or LeuGlyGly266-268GlyGlyAla substitution of the human AT, or the deletion of exon 3 or 4 of the human AT mRNA resulted in the generation of proteins with the FS activity^16,24^. We have also shown that the Met69Ser/Thr substitution or exon 3 or 4 deletion may have a synergistic effect to enhance the FS activity when combined with the LeuGlyGly266-268GlyGlyAla substitution, possibly due to the changes in protein conformation/localization by the former and the changes in sugar specificity by the latter. The present study demonstrated that the human ATs with any other amino acid residues than Ser or Thr at codon 69 also manifest FS activity. The activity also increased by combining with the LeuGlyGly266-268GlyGlyAla substitution. The use of newly generated COS1(B3GALNT1 + A4GALT) cells has drastically improved the sensitivity of FS activity detection. The AT construct with methionine also showed FS activity although it was the lowest among all the amino acid substitution constructs at codon 69. The conserved methionine residue at the corresponding positions in a variety of species in the *ABO* gene-encoded proteins, but not in the *GBGT1* gene-encoded proteins, seems to support the hypothesis that this substitution to methionine may have contributed to the separation of *ABO* genes from the *GBGT1* genes during the gene evolution after duplication.

There were several distinctions in the experimental procedures between immunocytochemistry and cytometry. Cells were fixed 3 days after DNA transfection in the immunocytochemical detection, whereas cells were immunostained directly without fixation 2 days after DNA transfection in the cytometry. The primary and secondary antibodies were used in the former, whereas an additional fluorescent dye (AF647)-tagged Streptavidin was used in the latter. However, the signal was also amplified by the peroxidase mediated enzymatic reaction in the former, which may have saturated signals with several constructs in the immunocytochemistry experiments.

The results reminded us of the importance of precursor substrate availability in analyzing the glycosyltransferase activity. It was evident in the *in vitro* enzymatic assays where different quantity of substrates may be included in the reactions. However, it has also proven to be correct even in cellular assays employing DNA transfection. As opposed to peptide/protein antigens of RBCs, glycan antigens are not primary gene products. Rather, they are the enzymatic reaction products catalyzed by specific glycosyltransferases. In the Michaelis–Menten kinetics, the reaction velocity υ (rate of formation of product [P]) is proportional to the concentration of a substrate [S] as indicated by the Michaelis-Menten equation: υ = d[P]/dt = V_max_[S]/(K_M_ + [S]), where V_max_ represents the maximum rate achieved at saturating substrate concentration and the Michaelis constant K_M_ is the substrate concentration at which the reaction rate is half of V_max_. Therefore, in the glycosylation reactions to produce RBC glycan antigens, the reaction velocity is dependent on the substrate concentration, whereas the V_max_ and K_M_ values are unique to individual enzymes. Our results demonstrate that the ordinary human AT may synthesize FORS1 under certain conditions where a sufficient concentration of Gb4 substrate is achieved. This may suggest that the FS activity may also be induced of the human AT when intra-Golgi concentration of another substrate, donor nucleotide sugar UDP-GalNAc, is elevated. In addition to the deletion of exon 3 or 4 of the human AT mRNAs we previously identified, the altered availability of donor and/or acceptor substrates may also account for an additional molecular mechanism that enables the human AT to express genetically inappropriate heterophilic FORS1 antigen in cancer cells/tumor tissues of ordinary individuals.

Gb3 and Gb4 are also called as P^k^ and P, and those expressed on RBCs are antigens of the P blood group system. α1,4-galactosyltransferase (Gb3 synthase, P^k^ synthase) encoded by functional *A4GALT* gene catalyzes the conversion of LacCer to Gb3 (P^k^). When this gene is mutated by the C > G substitution at nucleotide 631 resulting in the Gln to glutamic acid (Glu) substitution at codon 211, the α1,4-galactosyltransferase specificity is altered, and a rare inheritable NOR polyagglutination syndrome is manifested^27,28^. Additional Galα1-4-, GalNAcβ1-3Galα1-4-, and Galα1-4GalNAcβ1-3Galα1-4- structures may be linked to the non-reducing terminal GalNAc residue of Gb4, and globoside derivatives, NOR1, NOR_int_, and NOR2, are synthesized, respectively^29,30^. In spite of the fact that additional bands appeared in TLC analysis of nGSLs isolated from COS1(B3GALNT1 + A4GALT) cells, the *A4GALT* gene cDNA we used to generate those cells had an identical sequence to the reference NM_017436.4. Accordingly, those bands may not represent such Gb4 derivatives.

In this study we have demonstrated the FS activity of the human AT and its codon 69 substitutions constructs by improving the Gb4 substrate availability and the detection sensitivity. However, it should be mentioned that the enhancement is rather artificial. Several dozens of different types of tissues and 200 to 400 distinct types of cells are present in the human body. And individual cells/tissues may manifest unique expression profiles of glycosyltransferases and/or glycosidases. Accordingly, the results would have been entirely different if hematopoietic cells were used, in place of epithelial cells of COS1 cells and HeLa cells, in the experiments. There are other unknowns. For instance, human RBC membranes contain globoside as the prevalent neutral glycosphingolipid. Therefore, Gb4 is present at high concentration. In blood group A and AB individuals the AT should be present and functional during erythropoiesis leading to RBC differentiation because it produces cell-surface A antigens. Nonetheless, no FORS1 is synthesized. Accordingly, there may also be molecular mechanism(s) inhibiting the FORS1 biosynthesis in RBCs in FORS1-negative individuals. Such inhibitory mechanisms may need to be elucidated by future studies.

## Methods

### Preparation of human AT amino acid substitution constructs at codon 69 by *in vitro* mutagenesis

We performed the 2-round PCR using specific primers to introduce amino acid substitutions as previously described^13,15,24^. We used, as templates, H_ABO-A, human AT cDNA expression construct prepared in the pSG5 eukaryotic expression plasmid vector, and its derivative, H_ABO-A(GlyGlyAla), possessing the LeuGlyGly266-268GlyGlyAla tripeptide substitution. The detailed protocols are presented in the Supplemental Information.

### Generation of COS1(B3GALNT1 + A4GALT) cells

COS1(B3GALNT1 + A4GALT) cells were created from COS1(B3GALNT1) cells expressing human *B3GALNT1* gene-encoded β1,3-*N*-acetyl-d-galactosaminyltransferase 1^15,24^. We employed the glycan customization technology by modular expression of glycosyltransferases, utilizing the retroviral pMigR1g-A4GALT construct^31^. This construct available in the laboratory contained the human *A4GALT* gene cDNA followed by an internal ribosome entry site (IRES) element and then cDNA encoding enhanced GFP in the pMigR1g retroviral vector. DNA was transfected into Phoenix-AMPHO packaging cells, using Lipofectamine 2000 reagent (Thermo Fisher Scientific) following the manufacturer’s instructions. The culture media containing the viral particles were recovered and filtered. COS1(B3GALNT1) cells were then infected with the viral particles and allowed to grow to confluence. After being detached with trypsin/EDTA and re-suspended in culture media, cells expressing both the coupled blue and green emitting fluorescent proteins (mTagBFP2 and GFP) were FACS-sorted to generate COS1(B3GALNT1 + A4GALT) cells. The selected cells were expanded and used as recipients of DNA transfection.

### Isolation and TLC analysis of neutral glycosphingolipids

Neutral glycosphingolipid (nGSL) fractions were isolated using the protocol modified from “Extraction of glycolipids by Okino and Ito” in the GlycoPOD GlycoScience Protocol Online Database [https://jcggdb.jp/GlycoPOD/protocolShow.action?nodeId=t4]. Briefly, cells were grown in 4 plates of 15 cm cell culture dishes. When the cells reached confluence, they were PBS-washed, scraped, and harvested. Cell pellets were pooled for each sample by centrifugation. Lipid extraction was achieved by incubation with chloroform/methanol (2:1 v/v), sonication for 5 min in a sonicator bath (Selecta), followed by incubation at 37 °C for 1 h with agitation. After centrifuging (1000 × g, room temperature, 15 min), the solvent was transferred into a glass tube. The pellet was further extracted with chloroform/methanol/water (1:1:0.8) at 37 °C for 2 h with agitation. After centrifuging, the solvent was pooled with the first one. The total lipids extracted were dried by evaporation at 55 °C and resuspended in 4 ml of methanol. Saponification was performed by adding 50 µl of 4 M NaOH and incubating the sample at 37 °C for 2 h followed by neutralization with 50 µl of 4 M acetic acid. Then, 4 ml of water were added and the mixture was loaded onto Oasis H1B purification columns (reverse-phase cartridge, Waters Corp). After washing with methanol/water (1:1) and water, the glycosphingolipid fraction was eluted using methanol. The glycolipids were again dried by evaporation and resuspended in 4 ml of methanol followed by the addition of water. The resulting mix was loaded onto Oasis MAX columns (anion exchange cartridge, Waters Corp.). After methanol/water (1:1) and water washes, nGSLs were eluted with methanol whereas acidic GSLs (containing sialic acid) remained bound to the column. After drying, the glycolipids were resuspended in chloroform/methanol (1:1).

20 μl of nGSLs from COS1, COS1(B3GALNT1) and COS1(B3GALNT1 + A4GALT) cells were applied onto a TLC aluminum foil plate, together with the mixture of 2.5 μg each of lactosylceramide, globotriaosylceramide and gangliotetraosylceramide (asialo-GM1)(all from Sigma) on the “Standards” lane. After 30 min, the plate was placed in a chromatographic chamber containing chloroform/methanol/water (65:35:8). The ascending solvent separated lipids. When the front reached approximately 1 cm to the edge, the plate was retrieved from the chamber and allowed to dry. The plate was then sprayed with orcinol (Sigma) diluted in sulfuric acid and heated at 110 °C until purple bands became visible.

We performed direct immunostaining of nGSLs separated on TLC plate. 10 μl each of nGSLs from COS1(B3GALNT1 + A4GALT) and COS1(B3GALNT1 + A4GALT + mGBGT1) cells were applied and chromatographed as explained above. After drying, the plate was blocked and hydrated with 5% non-fat dry milk in Tris-buffered saline plus 0.05% Tween 20 (TBST) for 16 h. It was then incubated with clone FOM-1 rat IgM monoclonal anti-FORS1 antibody (Bio-Rad Antibodies) diluted at 1/1000 in the same solution for 3 h at room temperature. Following the TBST washes, the plate was incubated with rabbit anti-rat IgG + IgM + IgA H&L (HRP) antibody (Abcam) at 1/10000 dilution for 1 h at room temperature. It was then washed and incubated with ECL^TM^ Detection Reagents (Sigma). After exposure, the chemiluminescence-sensitive film (BD) was developed using an automated FUJI Medical Film FPM100A processor.

### Immunocytochemical detection of FORS1 antigen expression after transient DNA transfection of COS1(B3GALNT1 + A4GALT) cells and of blood group A antigen expression after DNA transfection of HeLa(FUT2) cells

Newly generated COS1(B3GALNT1 + A4GALT) cells were used as recipients of DNA transfection as previously described with COS1(B3GALNT1) cells^13,15^. Briefly, DNA from the AT or its mutant constructs and DNA from the pEGFP plasmid vector expressing enhanced GFP were co-transfected, using Lipofectamine 3000 reagent (Thermo Fisher Scientific). Three days after transfection, GFP-positive (GFP+) cells were counted under the fluorescence microscope to estimate the variations in transfection efficiency. The transfected cells were dried, fixed with 4% paraformaldehyde in PBS, washed, and then subjected to immunostaining. Clone FOM-1 rat IgM monoclonal anti-FORS1 antibody was used as the primary antibody to detect the FORS1 expression. The cells were next treated with a biotinylated goat anti-rat IgG + IgM(H + L) secondary antibody (Jackson ImmunoResearch Laboratories), PBS washed, and then incubated with the Vectastain ABC reagents (Vector Laboratories). 3,3′-diaminobenzidine tetrahydrochloride (DAB) from the same company was used for peroxidase-mediated color development. DAB-positive cells were counted under the microscope and the numbers were normalized using the numbers of GFP+ cells. The experiments were repeated 6 times for all the constructs, and the averages and standard deviations were calculated.

HeLa(FUT2) cells, HeLa derivative cells expressing transduced human *FUT2* gene cDNA encoding α1,2-fucosyltransferase (H enzyme) to increase H substance, the acceptor substrate for AT/BT, were also used as recipients of DNA transfection as previously described^13,15^. In addition to DNA from the expression constructs, DNA from the pEGFP plasmid was co-transfected, using Lipofectamine 3000 reagent. To detect A antigen expression, cells were immunostained with the mixture of murine anti-A monoclonal antibodies (BioClone, Ortho Clinical Diagnostics) as primary antibodies, and biotinylated goat anti-mouse IgM secondary antibody (Vector Laboratories). Otherwise, the same immunocytochemical protocols described above were employed.

To take the microphotographs of immunostained cells after DNA transfection, COS1(B3GALNT1 + A4GALT) and HeLa(FUT2) cells were seeded on 8-cell Millicell EZ slides (Merck Millipore) previously coated with 10 μg/ml poly-L-lysine (Merck). When cells reached to 70–90% confluence, they were transfected using Lipofectamine 3000. 3 days after transfecton, cells were fixed with 4% paraformaldehyde, rinsed with PBS, and immunostained as described above. After color development, DAB was removed, and cells were washed with water and PBS. The incubation chambers were removed, and the slides were treated with 95% ethanol and air-dried. The slides were mounted with Vectamount medium (Vector Laboratories) and then with glass coverslips (Erie Scientific Company). Cells were imaged using a DMI6000B microscope equipped with color camera DFC420 and LAS X software (Leica). Images were corrected for brightness and contrast using Adobe Photoshop CS5, and figures were generated using Adobe Illustrator CS5.

### Transient DNA transfection of expression constructs, FORS1 immunostaining, and cytometry of COS1, COS1(B3GALNT1), and COS1(B3GALNT1 + A4GALT) cells

COS1, COS1(B3GALNT1) and COS1(B3GALNT1 + A4GALT) cells were transfected with DNA from the selected expression constructs, using Lipofectamine 3000. DNA from pLL3.7-mRFP plasmid vector encoding mRFP was co-transfected to be used as efficiency control marker. After 48 h, cells were detached from culture plates with 5 mM EDTA in PBS and subjected to immunostaining, first with the primary FOM-1 anti-FORS1 antibody, then with the biotinylated goat anti-rat IgG + IgM(H + L) secondary antibody, and finally with AF647-conjugated Streptavidin (Thermo Fisher Scientific). A BD LSRFortessa Analyzer (BD Biosciences) was used for cytometry, employing the same settings for a single set of experiments. Viable single cells were gated based on their forward and side scattering values. Positive fluorescence of mTagBFP2-encoded blue fluorescent protein (BFP) was also used for gating the COS1(B3GALNT1) cells, whereas positive fluorescence of GFP and BFP was used for selecting the COS1(B3GALNT1 + A4GALT) cells. Fluorescence was measured at 575 nm to identify co-transfected mRFP expression and to determine the transfection efficiency. FORS1 expression was detected by AF647 fluorescence at 660 nm. Experiments were repeated three times, and FORS1+ cell percentages were calculated.

## Supplementary information

Supplemental Information

## Data Availability

The original data that support the findings of this study are available from the corresponding author on reasonable request (fyamamoto@carrerasresearch.org).
