# Supplementary material for: ABO blood group A transferase and its codon 69 substitution enzymes synthesize FORS1 antigen of FORS blood group system
Source: Sci Rep. 2019 Jul 4;9:9717. doi: 10.1038/s41598-019-46029-7 (PMC6609624; doi:10.1038/s41598-019-46029-7)
Supplement: Supplementary file 1 — Supplemental Information [file 41598_2019_46029_MOESM1_ESM.docx]

**Supplemental Information**

***Title:***

ABO blood group A transferase and its codon 69 substitution enzymes synthesize FORS1 antigen of FORS blood group system

***Authors:***

Miyako Yamamoto^1,#^, Maria Cristina Tarasco^1,2,#^, Emili Cid^1,3,#^, Hidetomo Kobayashi^1,4^, and Fumiichiro Yamamoto^1,3,*^

^#^ M.Y., M.C.T., and E.C. contributed equally to this work.

^*^To whom correspondence may be addressed.

ORCID identifiers:

<https://orcid.org/0000-0001-9516-1402> (MY), <https://orcid.org/0000-0002-5025-352X> (EC), <https://orcid.org/0000-0001-9690-7034> (FY)

***Affiliations:***

^1^Laboratory of Immunohematology and Glycobiology, Josep Carreras Leukaemia Research Institute (IJC), Campus Can Ruti, Camí de les Escoles, Badalona, Barcelona, 08916, Spain; ^2^Biologia Molecolare, Università degli Studi di Parma, Parma, 43121, Italy; ^3^Program of Predictive and Personalized Medicine of Cancer (PMPPC), Institut d'Investigació Germans Trias i Pujol (IGTP), Campus Can Ruti, Camí de les Escoles, Badalona, Barcelona, 08916, Spain; ^4^Laboratory of Molecular Microbiological Science, Faculty of Pharmaceutical Sciences, Hiroshima International University, Kure, Hiroshima 737-0112, Japan.

***Correspondence:***

Fumiichiro Yamamoto, Ph.D.

Josep Carreras Leukaemia Research Institute (IJC),

Can Ruti Campus, Ctra. de Can Ruti, Camí de les Escoles s/n, Badalona, Barcelona, 08916, Spain

E-mail: [fyamamoto@carrerasresearch.org](mailto:fyamamoto@carrerasresearch.org), Tel: (+34) 93 557 2820, Fax: (+34) 93 465 1472

**Keywords:**

ABO and FORS blood group system, blood group A transferase and Forssman glycolipid synthase (FS), blood group A, B, and FORS1 glycan antigens, glycosyltransferase specificity and activity

**Experimental protocols**

***In vitro* mutagenesis to prepare human AT amino acid substitution constructs at codon 69**

We used, as templates, H_ABO-A, human AT cDNA expression construct prepared in the pSG5 eukaryotic expression plasmid vector, and its derivative, H_ABO-A(GlyGlyAla), possessing the LeuGlyGly266-268GlyGlyAla tripeptide substitution. We performed the two-round primer-mediated polymerase chain reactions (PCRs) using specific primers to introduce amino acid substitutions as previously described.^11,13,22^

In the first approach of *in vitro* mutagenesis we used degenerate oligonucleotide primers containing NNN at codon 69 (Primers IJC-1 (sense) and -2 (antisense)) for PCR. The “N” stands for the equimolar mixture of G, A, T, and C nucleotides. All the 64 (4x4x4) triplets were assumed to be produced at a similar frequency. In the initial round of PCR, the translation initiation forward primer with an artificial *EcoRI* site (IJC-3) and the reverse primers around codon 69 (IJC-2) were combined to amplify DNA fragments encoding the *N*-terminal portions of the AT proteins, whereas the forward primers around codon 69 (IJC-1) were separately combined with the translation termination reverse primer with an artificial *BamHI* site (IJC-4) to amplify the *C*-terminal portion of the AT proteins. The nucleotide sequences of those primers are as follows.

IJC-1: 5’-CTCGTTGCCAAGG**NNN**GTCTACCCCCAGCC

IJC-2: 5’-GGCTGGGGGTAGAC**NNN**CCTTGGCAACGAG

IJC-3: 5’-CCCGGAATTCC**ATG**GCCGAGGTGTTGCGGA

IJC-4: 5’-CCCGGGATCCGC**TCA**CGGGTTCCGGACCGC

After PCR, both the reaction products were mixed and the second round of PCR was performed. The reaction products were then column-purified, digested with *EcoRI* and *BamHI*, column-purified, and ligated with pSG5 eukaryotic expression vector similarly digested with *EcoRI* and *BamHI* and further dephosphorylated to block self-ligation. The DNA was later used to transform frozen competent *Escherichia coli* bacteria. Bacteria from several dozens of individual transformant clones were grown, and plasmid DNA was isolated. DNA was then cleaved with *EcoRI* and *BamHI*, and the clones containing an AT cDNA were selected and sent for nucleotide sequencing at the GATC (currently named as Eurofins Genomics).

In the second approach of *in vitro* mutagenesis, more internal primers were designed to introduce specific amino acid substitutions, which were not obtained in the first approach, into the human AT. The same experimental protocols described above were also employed except for the primers themselves.

**Results**

**Forty human ATs with any one of the 20 amino acids at codon 69 with and without the LeuGlyGly266-268GlyGlyAla substitution were prepared.**

In the initial stage of *in vitro* mutagenesis using the degenerate oligonucleotide primers containing NNN at codon 69, we intended to obtain all the AT constructs with and without LeuGlyGly266-268GlyGlyAla substitution, containing any one of the 20 amino acids at that position. However, we did not obtain all. We actually obtained the AT constructs containing Ala, Arg, cysteine (Cys), Gln, Gly, histidine (His), isoleucine (Ile), Leu, lysine (Lys), Met, phenylalanine (Phe), proline (Pro), Ser, Thr, tyrosine (Tyr), or valine (Val) at codon 69, but failed to obtain those containing asparagine (Asn), aspartic acid (Asp), glutamic acid (Glu), or tryptophan (Trp) at the position. We also obtained the AT constructs with the LeuGlyGly266-268GlyGlyAla substitution, containing Arg, Asn, His, Leu, Met, Phe, Pro, Ser, Thr, Trp, or Tyr at codon 69, but failed to obtain those containing Ala, Asp, Cys, Gln, Glu, Gly, Ile, Lys, or Val.

Using the internal primers more specific to certain amino acid(s), the second approach of *in vitro* mutagenesis was repeated until we finally generated all the amino acid substitution constructs of the human AT containing any amino acid at codon 69 with and without the LeuGlyGly266-268GlyGlyAla substitution. We also obtained the constructs with the translation termination codon (Ter) at that position.
